# Downscaling global ocean climate models improves estimates of exposure regimes in coastal environments

**DOI:** 10.1038/s41598-020-71169-6

**Published:** 2020-08-26

**Authors:** Matheus Fagundes, S. Y. Litvin, F. Micheli, G. De Leo, C. A. Boch, J. P. Barry, S. Omidvar, C. B. Woodson

**Affiliations:** 1 School of Environmental, Civil, Agricultural, and Mechanical Engineering, University of Georgia, Athens, GA 30602 USA; 2 Department of Marine Sciences, University of Georgia, Athens, GA 30602 USA; 3 Monterey Bay Aquarium Research Institute, Moss Landing, CA USA; 4 Hopkins Marine Station, Stanford University, Pacific Grove, CA USA; 5 Stanford Center for Ocean Solutions, Pacific Grove, CA USA

**Keywords:** Climate-change ecology, Climate-change impacts

## Abstract

Climate change is expected to warm, deoxygenate, and acidify ocean waters. Global climate models (GCMs) predict future conditions at large spatial scales, and these predictions are then often used to parameterize laboratory experiments designed to assess biological and ecological responses to future change. However, nearshore ecosystems are affected by a range of physical processes such as tides, local winds, and surface and internal waves, causing local variability in conditions that often exceeds global climate models. Predictions of future climatic conditions at local scales, the most relevant to ecological responses, are largely lacking. To fill this critical gap, we developed a 2D implementation of the Regional Ocean Modeling System (ROMS) to downscale global climate predictions across all Representative Concentration Pathway (RCP) scenarios to smaller spatial scales, in this case the scale of a temperate reef in the northeastern Pacific. To assess the potential biological impacts of local climate variability, we then used the results from different climate scenarios to estimate how climate change may affect the survival, growth, and fertilization of a representative marine benthic invertebrate, the red abalone *Haliotis rufescens*, to a highly varying multi-stressor environment. We found that high frequency variability in temperature, dissolved oxygen (DO), and pH increases as pCO_2_ increases in the atmosphere. Extreme temperature and pH conditions are generally not expected until RCP 4.5 or greater, while frequent exposure to low DO is already occurring. In the nearshore environment simulation, strong RCP scenarios can affect red abalone growth as well as reduce fertilization during extreme conditions when compared to global scale simulations.

## Introduction

With ocean temperatures expected to continue to rise, and dissolved oxygen (DO) and pH to decrease, due to climate change, there is an urgent need to understand how marine ecosystems will respond to future ocean conditions^1–3^. While the biological impacts of future variability in these, and other, co-occurring environmental drivers are not well understood, the few studies available suggest that understanding variability and covariation of multiple environmental stressors are important for predicting organism responses in marine ecosystems^4,5^. Currently, climate model predictions are generally global or regional in scale. However, the consequences of future environmental variability on species and ecological processes are predicated on local exposure regimes, which often exhibit higher temporal and spatial variability^6–9^ relative to global estimates. Therefore, there is a critical need to downscale global forecasts to biologically relevant local scales.

Global climate models (GCMs) predict increases in surface ocean temperature of up to 4 °C, declines in oxygen of up to 0.78 mg L^−1^, and reduction in pH of up to 0.35 units by 2100^10–12^. One approach to understand how future global or regional predictions may manifest at local scales is to obtain higher spatio-temporal resolution estimates from GCMs through downscaling^13^. For example, downscaling of GCMs has been applied to resolve mesoscale oceanographic features. Using this approach, Busecke et al*.*^14^ recently showed that resolving mesoscale features (i.e. eddies) lead to a better representation of Equatorial Undercurrent (EUC) dynamics and the tilt of the Oxygen Minimum Zone (OMZ) in the Pacific, compared to 1° resolution results from a GCM. Downscaling has also been applied to understand how oceans will be affected by climate change at regional scales. In the California Current system (CCS)^15^, studies using downscaling approaches suggest by the year 2050 aragonite saturation state will decrease by 0.5–1 and the flux of nutrients, such NO_3_ (0.2 mmol-N m^−3^) and Si(OH)_4_ (0.8 mmol-Si m^−3^), will increase^15,16^. However, uncertainty in the ecosystem response in the CCS region remains due to the complexity of the environment, especially in nearshore ecosystems.

In the nearshore, tides, winds and waves (surface and internal) drive ocean dynamics^9,17,18^ at relatively small, short scales (i.e. < 1 km, < 24 h). Models that capture only mesoscale processes, and ignore local drivers, cannot resolve the dynamics of environmental conditions at biologically relevant spatial or temporal scales. For example, short term fluctuations in temperature, pH and DO that reach 5 °C, 0.5 pH units and 4 mg L^−1^ respectively at ~ 15 m depth in a ~ 13-h cycle (M2 tide) have been observed in Monterey Bay, CA, likely due to tidal forcing^7^ (Supplementary Fig. 1). In this system, nonlinear internal waves play an important role by advecting colder, low oxygen, and low pH waters into coastal ecosystems^8,9^, while large kelp forests contribute significant oxygen production and respiration that can alter local DO and pH dynamics^19,20^. However, current mesoscale oceanographic models that include Monterey Bay, CA do not capture these dynamics. Consequently, extrapolating future climate change scenarios to nearshore systems without simulation of high-frequency coastal processes such as diurnal winds, internal waves, tides, and consideration of biological process in the presence of this variability could drastically misrepresent associated ecosystem responses.

**Figure 1:**
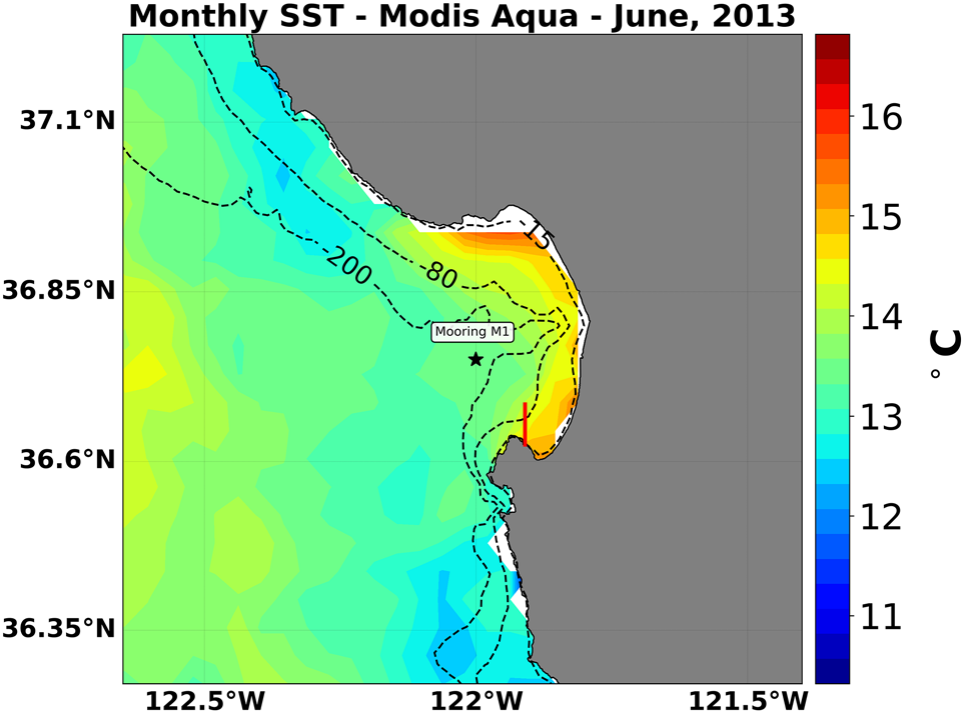
Monthly SST from MODIS-Aqua for Monterey Bay on June, 2013 showing warm water lenses near the coast of Monterey Bay, CA. Model domain based on the cross shore section (red line). Black star indicates the barotropic tides used for the model input and validation.

Adding to the complexity in predicting ecosystem responses under future climate scenarios; marine organisms often exist close to their tolerance limits and thus deviations of temperature, DO, and pH from natural background conditions can cause physiological stress^21^. Moreover, multi-stressor responses may differ from the aggregate of single stressor predictions, with both synergistic and antagonistic interactions frequently occurring^22^. For example, low DO and low pH act synergistically to reduce the survival and growth of juvenile red abalone (*Haliotis rufescens*)^23^, while increased temperature counteracts the negative impacts of low pH on fertilization rates in the same species^23^. Such interactions suggest that understanding how temperature, DO, and pH co-vary and how these conditions affect the local biota is critical for predicting biological responses to climate change^3,9,24,25^. Here, we show how variability in co-varying environmental drivers (T, DO, pH) may change under future conditions (increasing levels of CO_2_) and how these scenarios affect exposure of nearshore organisms to potentially stressful conditions. To do so, we developed and calibrated an idealized coupled biogeochemical hydrodynamic model using in situ data from the upwelling period in the region of southern Monterey Bay near Hopkins Marine Station, Pacific Grove, CA USA (Fig. 1). We then used a downscaling approach for Representative Concentration Pathway (RCP) scenarios for the year 2100 as boundary and initial conditions in the model and estimated the potential effect on growth and fertilization for the red abalone, *Haliotis rufescens*. *H. rufescens* was chosen because there is extensive experimental data available on the interactive effects of environmental stressors on their growth and fertilization. Red abalone also represent a variety of benthic calcifying marine invertebrates with a motile larval stage and sedentary post-settlement stages, and have important economic value^26^.

## Methods

### Study region

Monterey Bay is the largest bay on the California coast, and has a total area of approximately 1,162 km^2^ (Fig. 1). Due to upwelling, the bay is highly productive and supports dense kelp forests, dominated by *Macrocystis pyrifera*, on rocky reefs that extend to approximately 20 m depth^27^. These forests have the capacity to regulate pH^28–30^ and sustain over 200 different species, from phytoplankton to marine mammals^31^.

Temperatures are the coolest in Monterey Bay during spring and early summer due to wind-driven upwelling^32^, driven by equatorward winds blowing over the California coast causing offshore Ekman transport^33^. During the same months, filaments of water originating from Point Año Nuevo to the north are trapped within the Bay, where they warm due to solar radiation, forming a lens of warm water close to shore^34,35^ (Fig. 1). Furthermore, a weak cyclonic eddy is observed within the bay due to the coastal geometry^32,34^. Inside the bay, sea breezes and tides drive diurnal and semi-diurnal currents that can lead to significant variability in environmental conditions^17,36^. During the upwelling period, salinity is approximately 34, temperature ranges from 9 to 13 °C at 17 m depth^7^, DO varies from as low as 100 μmol kg^−1^ (3.2 mg L^−1^ for T = 13 °C, S = 34 at 17 m) to as high as 300 μmol kg^−1^ (9.62 mg L^−1^ for T = 13 °C, S = 34 at 17 m), and pH varies from 7.7 to 8.1^3^ (Supplementary Fig. 1).

The three primary barotropic tidal constituents in the region (M2, K1, and S2) are responsible for over 80% of the tidal amplitude observed^18^. In the southern region of the bay, tides are mainly responsible for the cross-shelf velocity^36^ and the interaction of these surface tidal currents with the steep topography create internal tides comprised of internal waves and bores occurring at tidal frequencies^37,38^. Internal tides are formed when currents move over steep slopes, dense waters are forced into shallower regions, and, as these waters sink back to depth, an internal wave is generated^39^. These internal waves have speeds^8^ on the order of 0.05–0.2 m s^−1^ and, as the coast steepens, can break forming internal bores that move upslope and bring cold, low DO, low pH waters into nearshore kelp forest ecosystems^40^. During upwelling, these processes drive variability in temperature, pH and DO over semi-diurnal and diurnal periods that can exceed the predicated changes in mean conditions predicted by global climate models for year 2100^41^.

### Model description

**Figure 2:**
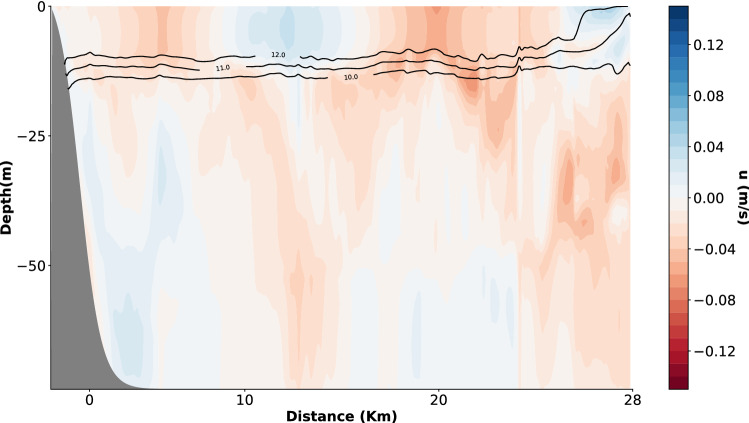
Snapshot of model crosshore velocity and temperature (contour lines) on 8 July 2013. Positive values are onshore, negative values are offshore. Contour lines (black) show isotherms with temperature labels.

To better understand how tides and winds affect exposure of nearshore organisms to variability in temperature, DO, and pH under current and RCP climate scenarios, we used dynamic downscaling and developed a 2D coupled biogeochemical hydrodynamic model using ROMS^42^. The model domain was created based on the Monterey Bay continental shelf described in Walter et al.^6^ with a maximum offshore depth of approximately 80 m (Fig. 2). The biogeochemical model used is described in Fennel et al.^43,44^. We forced the model with representative wind (diurnal sea breeze accounting for regional winds), solar radiation, and tidal currents for the Monterey Bay region (Supplementary Figs. 2–6; Supplementary Table 1). Detail of the full model structure, including boundary and initial conditions, are included in the “Supplementary Information”.

We estimated depth profiles for temperature, DO, and pH for the downscaled coupled hydrodynamic-biogeochemical model. We considered a homogeneous salinity (S = 34) for the entire domain since the variation in salinity is less than 0.4 over an entire year and less than 0.05 during the upwelling season in the Monterey Bay region^27^. Thus, initial and boundary stratification are assumed to be controlled by temperature alone. We represent initial (IC) and boundary conditions (BC) for temperature, DO, and pH using:

1 $$ {\text{var}} \left( {\text{z}} \right) = \left\{ {\begin{array}{*{20}l} {\Delta {\text{var}} \;{\text{D}}_{{{\text{pyc}}}}^{\alpha } } \hfill & {{\text{if}}\;{\text{z}} \ge {\text{D}}_{{{\text{pyc}}}} } \hfill \\ {\Delta {\text{var}} \;{\text{z}}^{\alpha } } \hfill & {{\text{otherwise}}} \hfill \\ \end{array} } \right. $$

where *var* is the identified variable (e.g. temperature), z is the depth, α is a fit coefficient for each variable determined from a least-squares fit to observational data, and D_pyc_ is the depth of the pycnocline. We used this method to estimate profiles of temperature, dissolved inorganic carbon (DIC), and DO for present and all downscaled future scenarios setting D_pyc_ to 17.5 m. The data used for the initial and boundary conditions (BC) of phytoplankton and chlorophyll profiles were taken from Schuckmann et al.^45^. The initial chlorophyll concentration was converted to zooplankton concentration (zoop = 0.34 × 10^–3^ mmol m^−3^) using Eq. 3 from Wiebe^46^, a method that has been used in other studies^47,48^. Detritus was initially set to zero in the entire domain. All biogeochemical variables were forced hourly at the southern boundary.

For each scenario, we estimated depth profiles for temperature, dissolved oxygen, and DIC using Eq. (1). In order to fit Eq. (1), we obtained our estimated values of each variable near the surface, at 80 m depth on the shelf during upwelling for Present and 200 m depth for Future, respectively. We used 200 m depth from the future data set as this is the most common depth of source waters for upwelling in the region^49^. Present surface and bottom values of temperature, DO, total alkalinity (TA), and DIC for present scenario were based on Koweek et al.^27^. The mean of the 3-month period of strong upwelling (May, June, and July) was used to obtain values of temperature (surface and depth), oxygen (surface), and DIC (surface) from Representative Concentration Pathway (RCP) for the year of 2100 from the 4th report of the IPCC^50^. Since only surface values for DO and DIC and no values for TA were available, we estimated values for these parameters at depth.

For DIC, we assumed that the ratio between surface and bottom values (80 m for Present and 200 m for Future) in present conditions will not change for future scenarios:

2 $$\frac{{DIC}_{Surf}^{Present}}{{DIC}_{Bottom}^{Present}}=\frac{2073}{2280}= 0.909$$

Therefore, to find the bottom values for future scenarios we divided RCP surface values by this ratio:

3 $$\frac{{DIC}_{Surf}^{RCP8.5} }{0.909}= \frac{2167 }{0.909} = 2384\;\mathrm{mmol\;C}\;{\mathrm{m}}^{-3}$$

**Figure 3:**
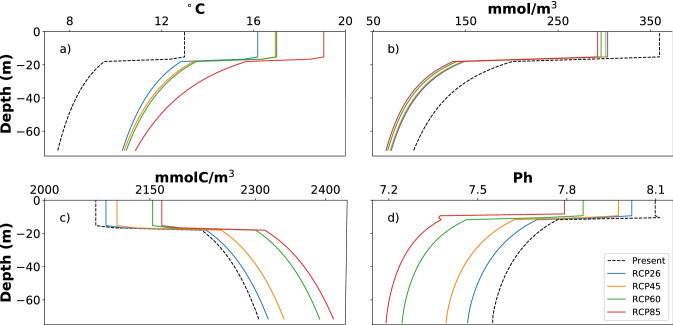
Initial and Boundary Conditions profiles for present and future scenarios: (**a**) temperature, (**b**) O_2_, (**c**) DIC, (**d**) pH.

Surface and bottom TA values were kept the same as present conditions, following Feely et al.^3^. For DO, bottom values for RCP 2.6 and 8.5 were approximated from Figs. 5 and 6 of Bopp et al*.*^51^. We calculated the ratios between surface and bottom DO for Present, RCP 2.6, and RCP 8.5. The ratio of surface:bottom DO was not constant across scenarios, so we approximated the ratios for RCPs 4.5 and 6.0 using linear least squares fit (Table 1; example calculation in the “Supplementary Information”). We, then applied the values from Table 1 in order to calculate α assuming D_pyc_ = 17.5 m. We then used Eq. (1) to generate the initial and boundary conditions (Fig. 3).

**Table 1:** Values for present (empirical data) and future (global ocean models) surface and estimated bottom conditions used to fit Eq. 3 and used as boundary conditions for the downscaled model runs.

Scenarios	Location	T (°C)	pCO_2_ (ppm)	O_2_ (mmol m^−3^)	O_2_ (mg L^−1^)	TA (mEqm^−3^)	DIC (mmolC m^−3^)	pH	Ω_ar_
Present	Surface	13.00	400	300	9.38	2,300	2,073	8.11	2.5
	Bottom	8.00		30	0.94	2,305	2,280	7.63	0.77
RCP 2.6	Surface	16.20	430	243.7	7.61	2,300	2,057	8.01	2.35
	Bottom	10.27		25	0.78	2,305	2,298	7.53	0.7
RCP 4.5	Surface	16.95	540	241.7	7.55	2,300	2,103	7.97	1.94
	Bottom	10.35		19.22	0.60	2,305	2,313	7.47	0.62
RCP 6.0	Surface	17.00	670	236.7	7.40	2,300	2,154	7.85	1.75
	Bottom	10.21		18.6	0.58	2,305	2,370	7.3	0.42
RCP 8.5	Surface	19.00	930	232.8	7.28	2,300	2,167	7.79	1.37
	Bottom	10.81		15	0.47	2,305	2,384	7.26	0.39

We calculated pH and Ω_ar_ using the CO2SYS^52^ package in MATLAB using temperature, salinity, DIC, and Total Alkalinity (TA) from the simulations at the offshore location where the bottom depth was 15 m. We assumed concentrations of phosphate and silica based on Koweek et al.^27^. We used dissociation constants for H_2_CO_3_ and HCO_3_ from Dickson and Millero^53^ and hydrogen sulfate ion constant (HSO^-4^) from Dickson^54^. All surface oxygen values were shifted positively 60 mmol m^−3^ (1.87 mg L^−1^ for T = 13 °C and S = 34) in order to simulate the high primary production due to kelp forests^29^, which is not specifically accounted for in the model. Overall, temperature at the bottom remained constant across all scenarios, with exception of RCP6.0 where temperature increased 0.2 °C (Supplementary Table 2).

### Integrated exposure

Field observations^24,55^ and laboratory experiments^5,56^ have shown that below sub-lethal thresholds marine organisms inhabiting nearshore marine habitats in upwelling systems exhibit signs of physiological stress when exposed to elevated temperatures, low oxygen levels, or low pH waters, which is especially detrimental especially for calcifying species. Exposure of organisms to stressful temperature, DO, and pH conditions (φ_th_ where φ refers to temperature, DO, or pH) was done by subtracting the threshold value for a given organism and life stage from the model or observational data at 15 m water depth, then setting all positive values to zero for pH and O_2_, and all negative values to zero for temperature. Next, we estimated integrated exposure (E_*int*_) by integrating absolute exposure over a period of a week with a window interval of 1 h:

$$ \emptyset^{\prime} = \emptyset - \emptyset_{th} \left\{ {\begin{array}{*{20}c} {\emptyset^{\prime} > 0 \to \emptyset^{\prime} = 0\;for\;pH\;and\;O_{2} } \\ {\emptyset^{\prime} < 0 \to \emptyset^{\prime} = 0\;for\;temperature} \\ \end{array} } \right. $$

4 $$ E_{int} = \mathop \int \limits_{0}^{t} \left| {\emptyset^{\prime}} \right|dt $$

Thresholds of temperature, dissolved oxygen and pH (16 °C^57^, 4.85^25^ mg L^−1^, and pH of 7.5^23^ respectively) representing non-interactive negative impacts on juvenile red abalone growth were based on literature values^5,23,57^. Integrated exposure quantified the time and degree of stress an organism experiences, similar to the degree heating week with units of ^o^C w, or day (^o^C d) measure used to estimate thermal stress on coral reefs^58^, and has been previously used to understand the exposure of juvenile abalone populations to similar stressors in an empirical field study^57^. Overall, red abalone threshold values chosen had a strong negative effect on the species^5,23,57^, therefore, we used E_*int*_ as a proxy for estimating the potential impact of future conditions on abalone growth and survival.

### Fertilization response

**Figure 4:**
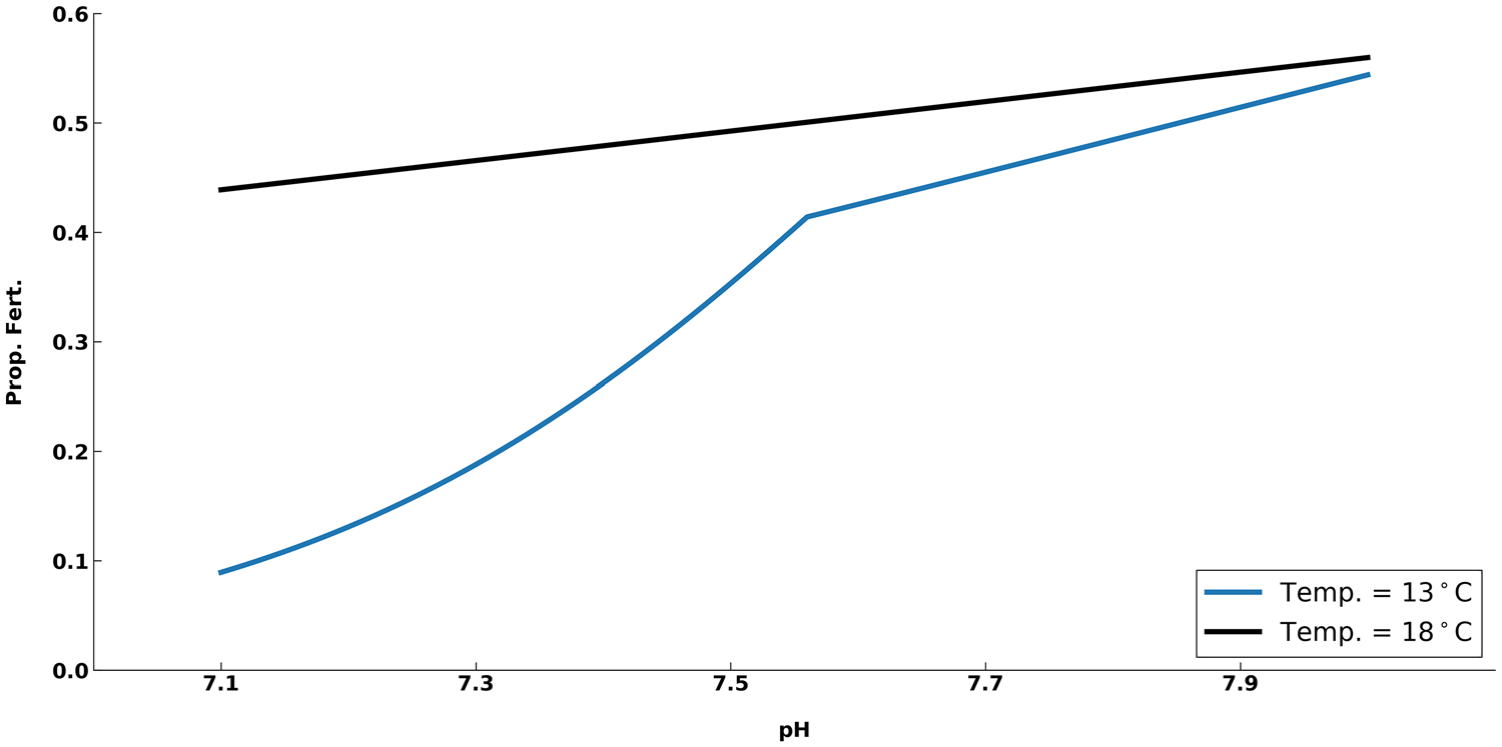
Proportional Fertilization (Prop. Fert.) as a function of pH for red abalone Eq. (5)—blue line; Eq. (6)—black line based on Boch et al.^25^ (Fig. 4a,c). For our study we used 15.5 °C as a transition between the two curves shown.

We estimated fertilization success using results of Boch et al.^25^ where the fertilization response of red abalone (*Haliotis rufescens*) was quantified in response to multiple stressor climate conditions (high temperature, low DO, and low pH). Fertilization in abalone occurs over relatively short periods, therefore E_*int*_ would not provide an appropriate estimate in such cases. While the process of fertilization occurs over short periods, adult red abalone exhibit an extended spawning season, over which environmental conditions may vary greatly based on our modeling results. Thus, we used the equations from Boch et al.^25^ to examine how fertilization success over a one-month period might be affected by environmental variability, specifically the interactive effects of ph and temperature. Changes in DO did not show a strong effect on fertilization in their experiments (Fig. 4; see Supplementary Table 3 for parameter values):

For temperatures = 13 °C:

5 $$ {\text{Logit}}\;\left( {\% Fert.} \right) = \left\{ {\begin{array}{*{20}l} {{\upbeta}_{0} + {\upbeta}_{{{\text{pH}}}} {\text{pH}}} \hfill & {{\text{for}}\;{\text{pH}} \le {\text{BP}}} \hfill \\ {\left( {{\upbeta}_{{{\text{pH}}}} + \left( {{\upbeta}_{2} - {\upbeta} } \right)} \right){\text{pH } - \text{ offset}}} \hfill & {{\text{for}}\;{\text{pH}} > {\text{BP}}} \hfill \\ \end{array} } \right. $$

For Temperatures = 18 °C:

6 $$\mathrm{Logit}\;(\mathrm{\%}Fert.)= {\upbeta }_{0} + ({\upbeta}_{\mathrm{p}H} + {\upbeta }_{\mathrm{A}})\mathrm{p}H + {\upbeta }_{\mathrm{B}}$$

where ${\upbeta }_{0}$ and ${\upbeta }_{\mathrm{pH}}$ are intercepts, $\upbeta$ and ${\upbeta }_{2}$ are slope segments, ${\upbeta }_{\mathrm{A}}$ is slope of the pH-temperature interaction (pH × Temperature Group), ${\upbeta }_{\mathrm{B}}$ is accounts for high temperature effects, and BP is the curve breaking point. Since only curves for 13 °C and 18 °C were available, we used 15.5 °C as a transition where Eq. (5) was applied for temperature less than 15.5 °C and Eq. (6) was applied for temperature greater than 15.5 °C. Since more complex interpolation schemes yielded similar results, we used this straightforward method for clarity.

### Model evaluation

We first assessed whether the oceanographic model was able to reproduce current (observed) oceanographic conditions in Monterey Bay. The model was expected to reproduce the main dominant semi-diurnal and diurnal periods of oscillations observed in the region as well as primary production regulation of DO and pH (excluding kelp) in the biogeochemical model. We had two requisites to consider the model performance satisfactory. First, we required that the model was capable of reproducing local minima for DO. Second, the model needed to be capable of reproducing the mean and extremes for temperature and pH. In addition, we anticipated observing lower values of DO in surface waters since we did not account for the higher primary productivity observed in kelp forests^20^, and therefore, DO oversaturation. To evaluate the biogeochemical model, we compared chlorophyll concentration in the model to satellite-derived chlorophyll estimates for the region. Monthly mean depth averaged chlorophyll by area (mg Chl m^−1^) was calculated for the model and for the months of May, June, and July from Sea-Viewing Wide Field-of-View Sensor (SeaWiFS)^59^ for the period of 2010–2017 to the closest region with data available next to our model simulation (cross section region in Fig. 1). Chlorophyll concentrations in the model were 2.12 mg Chl m^−3^ compared to 4.02 mg Chl m^−3^ estimated from SeaWifs. Thus, modeled values were within the range observed in the satellite data over this period.

**Figure 5:**
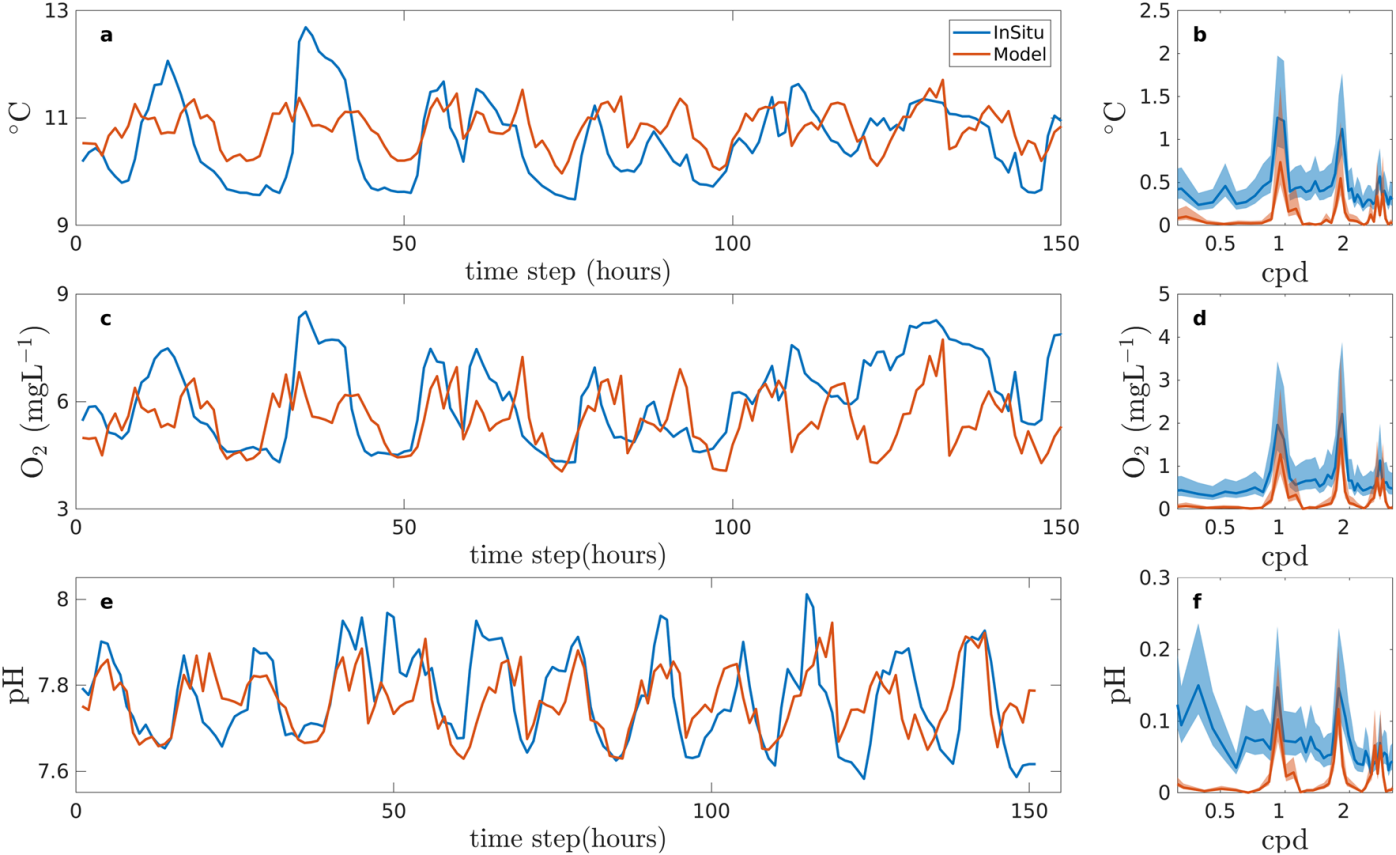
Time series for in situ^5^ and model data and power spectra of temperature (**a**,**b**), DO (**c**,**d**), and pH (**e**,**f**). Time series for the model was shifted to match tidal phase of the in situ data. *cpd* cycles per day.

In order to validate temporal variability in the model results, we estimated power spectra using the Thomson Multi-taper method (MTM)^60^. This method was chosen due to its robustness for stationary data with low variance. Power spectra allowed us to quantify the variability by frequency and confirm that the model was reproducing variability at dominant periods (M2, K1) observed in the region, as initial and boundary conditions in the model were based on regional observations. We applied the analysis over a 3-week window of upwelling for temperature, DO, and pH and compared with Booth et al.^7^ data (Fig. 5). Spectral analysis was used to ascertain the dominate temporal constituents of temperature, DO, and pH for all scenarios (present and RCP) between 12- and 24-h periods. The spectra were then integrated numerically to calculate the variability of temperature, DO, and pH, and the 95% confidence interval at each frequency band. Bootstrap analysis was applied to the integrated exposure calculation for each variable. In order to assure convergence in our estimates, 1,000 iterations with replacement were done using a sample size of 50% of the data. In the end, the confidence interval (CI) was calculated based on 2.5% percentile and 97.5% percentile of the distribution of the estimated means.

Our model results reflect current variability in temperature, DO, and pH in southern Monterey Bay (Fig. 5). In addition, mean temperature and pH values were not significantly different from in situ data. However, mean DO in our model was lower than present day averages, likely due to the lack of oxygen-producing kelp in our model^61^. Thus, the model accurately simulates diurnal, semi-diurnal, and higher order tidal components^62^. For Monterey Bay, diurnal (cycles per day (cpd) = 1) and semi-diurnal (cpd = 1.93) are the main temporal components of variability in temperature, oxygen, and pH and are well represented by our model with overlapping 95% confidence intervals (Fig. 5b,d,f). Higher frequency variability (cpd = 3) is also within the 95% confidence interval when comparing model and observations. However, frequencies occurring between peaks are not well resolved (Fig. 5b,d,f). We expect this is because we used a 2D model, and therefore, the model was unable to resolve all the physical processes occurring in the Monterey Bay. The oscillations between peaks in the observed data were likely due other coastal ocean processes such 3D circulation and ocean surface waves^36^ (as well as noise in the instruments). However, the variability at these periods did not have an appreciable effect on exposure calculations. Importantly, the model preserved the observed diurnal and semi-diurnal variability that is not present in global and regional scale climate models for future RCP scenarios (Fig. 5a,c,e).

### Variability at 15 m

The model was able to simulate the main drivers of variability in temperature, DO, and pH (predominantly internal waves) for the region in study. Internal waves in the domain were seen as vertical changes in the u-component of the velocity (Fig. 2). Cross-shelf velocities ranged from − 0.05 to 0.1 m s^−1^, and were within the range found in other studies^8,18,27^. Before the arrival of the internal wave crest, isotherms were tilted downwards indicating previous downwelling. Flow in opposite directions between crests was observed during retreating of internal waves in the domain, as it has been observed in studies on internal waves with in situ data^8,9^.

High variability in temperature, DO, and pH was observed in all model runs (Supplementary Figs. 7–9). Mean temperatures were 10.63 °C (SD = 0.39), 13.81 °C (SD = 0.46), 15.46 °C (SD = 0.50), 15.45 °C (SD = 0.49), 16.96 °C (SD = 0.95) for present, RCP 2.6, RCP 4.5, RCP 6.0, and RCP 8.5, respectively (Supplementary Fig. 7). Overall, mean temperature increased as expected and exhibited similar variability (standard deviation [SD]) across all RCPs except for RCP 8.5 scenario. This was likely due to increased temperature stratification where surface waters warm faster than deeper waters resulting in higher temperature variability.

Mean dissolved oxygen values were 5.15 mg L^−1^ (SD = 0.74) for present, 4.80 mg L^−1^ (SD = 0.88) for RCP 2.6, 5.32 mg L^−1^ (SD = 0.77) for RCP 4.5, 5.02 mg L^−1^ (SD = 0.81) for RCP 6.0, and 4.93 mg L^−1^ (SD = 1.21) for RCP 8.5 (Supplementary Fig. 8). The SD for RCP 8.5 was almost twice that of other scenarios. This was likely due to a stronger gradient in oxygen (surface remains saturated while the values at depth are lower). RCP 2.6 had the lowest mean DO but not different than the rest and RCP 4.5 was 0.52 mg L^−1^ higher, though not significantly different than the present scenario. The consequence for the lowest mean DO in RCP 2.6 was related to the strength of density stratification^61^, and therefore, low oxygen at 15 m. Another scenario (not shown) was used where RCP 4.5 oxygen profile was applied using the density stratification from RCP 2.6 and the same low oxygen values found previously were also observed in the alternate run, supporting this mechanism.

pH variability was also high across all model runs (Supplementary Fig. 9). The mean value for pH decreased from 7.73 (SD = 0.07) for present to 7.44 (SD = 0.12) for the RCP 8.5 scenario. RCP 8.5 again had the highest variability among all scenarios. Otherwise, the pH range was ~ 0.075 for all other scenarios. Lower pH for the most extreme scenarios (RCP 6.0 and 8.5) has also been observed in large scale models^12^.

## Results and discussion

**Figure 6:**
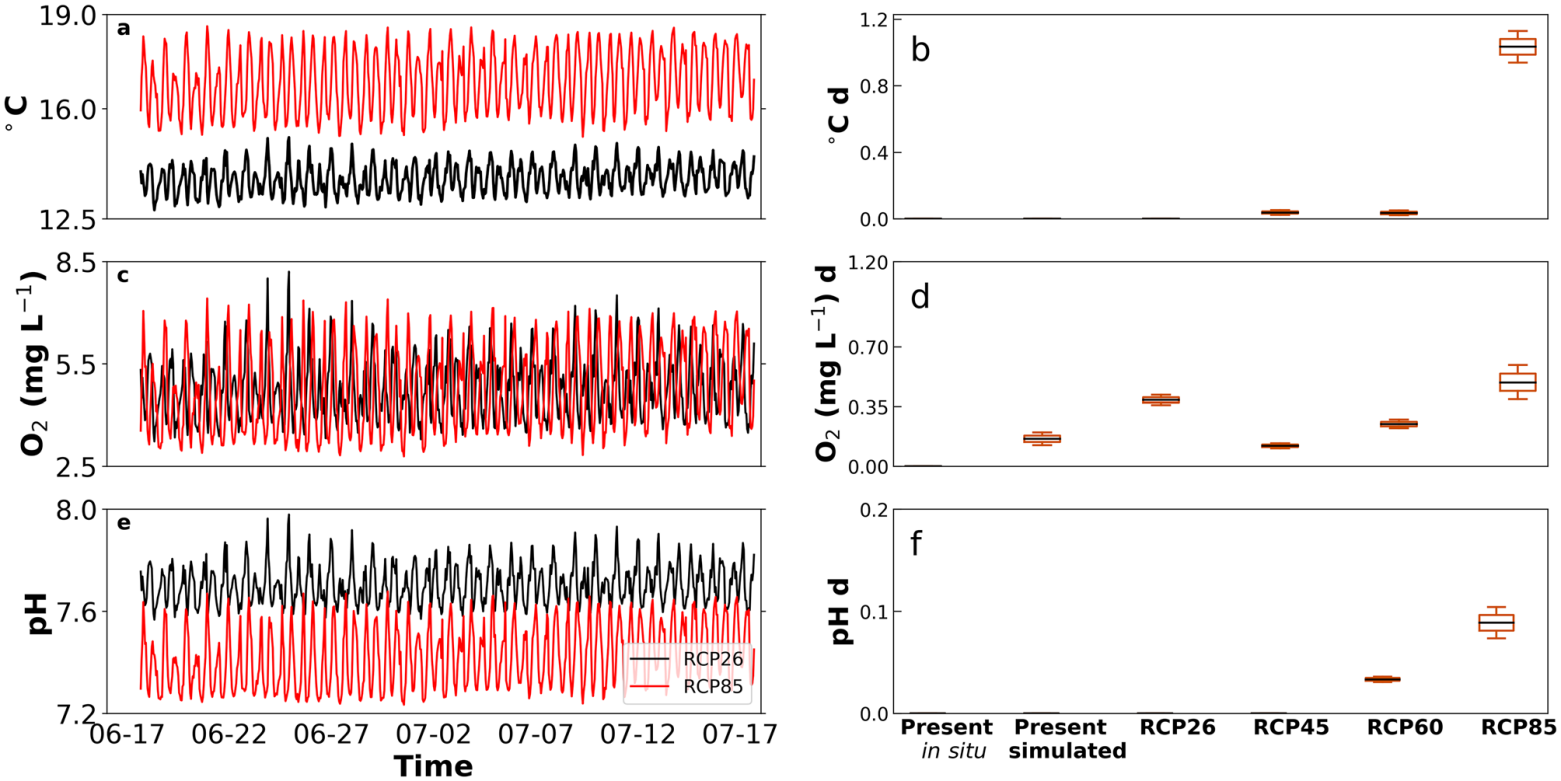
Modelled time series of (**a**) temperature, (**c**) DO, and (**e**) pH for 1 month period with idealized upwelling and boxplot of the mean daily integrated exposure to extreme (**b**) temperature, (**d**) dissolved oxygen, and (**f**) pH for present in situ, present simulated and future scenarios for red abalone (*Haliotis rufescens*).

**Figure 7:**
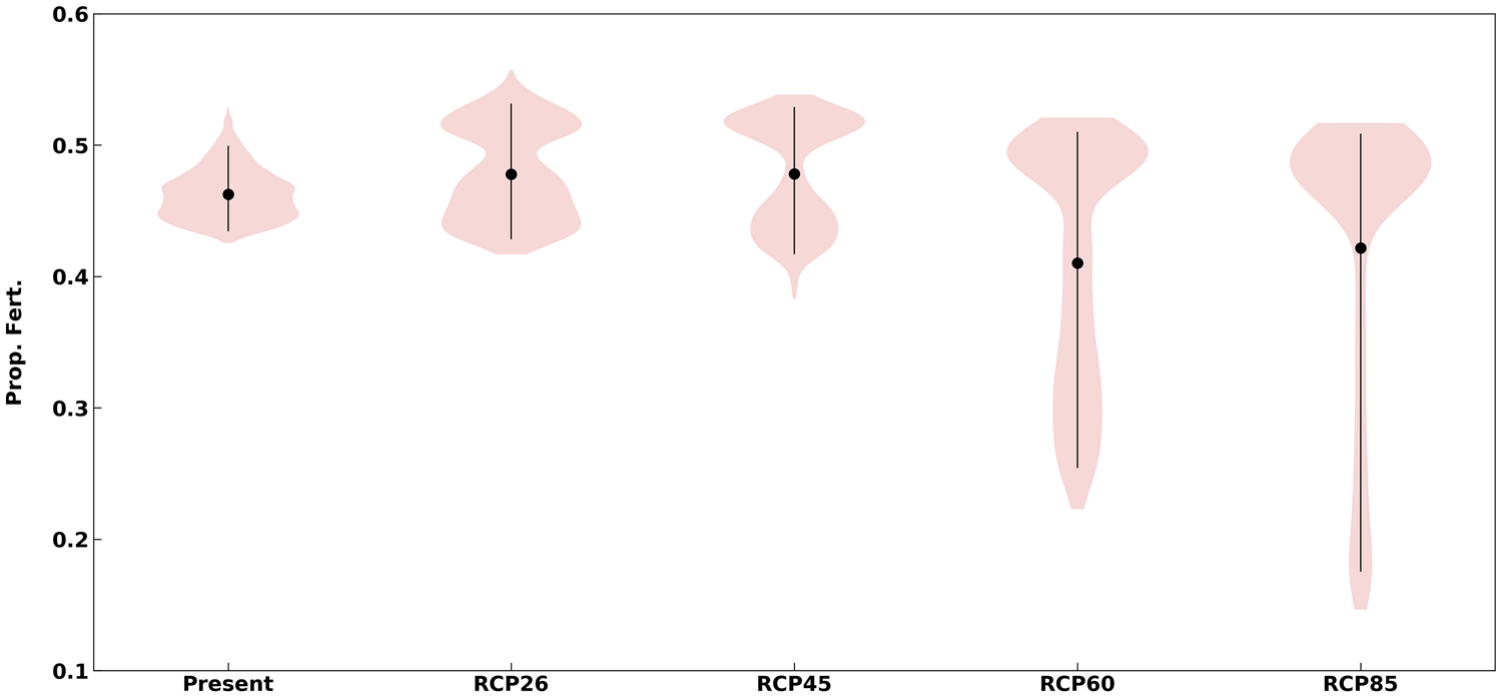
Red abalone fertilization response to multiple stressors for present and future RCP scenarios. Violin plot shows the distribution of proportional fertilization (Prop. Fert.) based on the threshold used. Black lines indicate 90% confidence interval, black dots show mean proportional fertilization.

Here, we focus on the estimated effects on growth (integrated exposure) and fertilization rates for red abalone (Figs. 6, 7). Integrated mean daily exposure (hereafter 'exposure') to potential stressors individually showed a positive trend from present to RCP 8.5 for all three environmental parameters (Fig. 6b,d,f). Exposure to Present in situ conditions showed similar exposure when compared to our simulated Present exposure with the only significant difference in the exposure to low DO. Exposure of juvenile red abalone to high temperature increased approximately linearly from none at present to 1.11 °C day in RCP 8.5. Similarly, exposure to low DO showed a trend across RCPs (Fig. 6d), where variability in mean daily exposure was highest (SD = 0.068 mg L^−1^ day) in RCP 8.5. Exposure to low pH was generally low except in the last two scenarios (0.106 and 0.166 pH d for RCP 6.0 and RCP 8.5, respectively) (Fig. 6f). Variability in pH exposure also increased over all RCPs, and was highest for RCP 8.5 scenario (SD = 0.020 pH day).

To quantify the interactive effects of multiple stressors on red abalone fertilization success, we used functional relationships from Boch et al*.*^25^ for temperature and pH to predict the proportion of fertilized gametes hourly over a 6-week period. Fertilization success (hereafter fertilization) showed wide variation across all RCP scenarios (Fig. 7). Mean fertilization rate only decreased for red abalone in RCP 6.0 and 8.5. This pattern was also observed for integrated pH exposure (Fig. 6f). For the present through RCP 4.5 scenarios, the pH was in the range of moderate fertilization success (pH > 7.5, Boch et al*.*^25^) and temperatures had little effect. In RCPs 6.0 and 8.5, pH was low enough (< 7.5) that often fertilization success was extremely low (< 0.25), however temperatures were often warm enough to keep fertilization in the same range of the other scenarios (0.4–0.5). Although we found that future changes in the environmental drivers could be stressful to abalone, higher temperatures often counteract the effect of pH on fertilization for red abalone^25^.

Present environmental variability in the nearshore can be larger than the total mean change predicted by large-scale climate models by the year 2100^41^. Using idealized tidal and wind patterns, our downscaled model captures the present observed variability in environmental drivers at diurnal and semi-diurnal frequencies in Monterey Bay, demonstrating the model’s ability to account for local processes which ultimately govern climatic variability at small scales. The importance of this downscaling approach in estimating potential biological impacts of future climate change is demonstrated by the comparing the predicted impacts on abalone fertilization and juvenile growth from our model versus those from the large-scale model, for a given RCP scenario (Table 2). Our results suggest that predicted impacts on red abalone growth and fertilization based on RCP 8.5 (non-upwelling) and RCP 8.5 (upwelling) are either under or overestimates, relative to the predictions based on the 2D model, demonstrating the need for downscaled models to estimate ecological impacts in nearshore systems under future climate change. The need for downscaled models is more important when considering the stress imposed on organisms due to frequent changes in conditions. For example, abalone may adapt to a constant elevated mean temperature, but not to rapidly fluctuating elevated temperatures.

**Table 2:** Estimates of exposure to temperature, DO, and pH stress for red abalone*, Haliotis rufescens*.

	Mean Temp (°C)	Mean O_2_ (mg L^−1^)	Mean pH	Prop. Fert.^a^	Total exposure^b^		
					°C day	mg L^−1^ day	pH day
CMIP5 (RCP8.5) (non-upwelling)	19.00	7.25	7.79	0.50	21.00	0.00	0.00
CMIP5 (RCP8.5) (upwelling)	10.81	2.00	7.20	0.29	0.00	26.04	2.10
2D model	17.00	4.93	7.44	0.40	6.00	5.19	0.85

^a^Proportional fertilization interpolated based on Figure 4 from Boch et al.^25^.

^b^Thresholds for red abalone used in order to calculate exposure for temp, DO, and pH.

In addition to demonstrating the importance of accounting for local processes in determining future climatic variability at small nearshore scales, this study highlights that future biological impacts may vary significantly across life stages. For red abalone fertilization is vulnerable to pH^25^, though this response is mediated by the ambient temperature. Therefore, fertilization in future climate scenarios could show either no effect or completely fail due to pH depending on the ambient temperature. In a future nearshore ocean with high environmental variability, timing of gamete release could be crucial in determining fertilization success. Benthic organisms such as abalone will experience effects on growth only in a few of the scenarios simulated using our downscaling approach (RCP 6.0, 8.5 for temperature, RPC 8.5 for DO, and 4.5 and on for pH), scenarios where elevated temperatures predicted by our downscale model could help ameliorate the negative impacts of pH on fertilization^23^.

Our study illustrates how the high temporal and short spatial scale oceanographic processes in the nearshore environments can have significant effects on the exposure of organisms to climate-related stressors. Our results also demonstrate that laboratory experiments investigating the effects of climate change scenarios on benthic organisms must include high frequency variability, both current and predicted future, and determine the interactive effects of environmental drivers in order to determine how marine ecosystems, and the organisms that comprise them will respond to future conditions. Our methodology could be (1) generalized to regions within other upwelling ecosystems with high diurnal and semi-diurnal variability^61,63^, (2) used to estimate future biological effects for species, beyond abalone, where environmental thresholds are established, and (3) applied to design experiments to identify species-specific thresholds to future in situ environmental conditions. However, the downscaled model assumes that ocean stratification, wind patterns, and biochemical rates will not change by the end of twenty-first century, and therefore, these predictions should be tested in future studies.

## Supplementary information

Supplementary Information.
