# Supplementary material for: Downscaling global ocean climate models improves estimates of exposure regimes in coastal environments
Source: Sci Rep. 2020 Aug 26;10:14227. doi: 10.1038/s41598-020-71169-6 (PMC7450070; doi:10.1038/s41598-020-71169-6)
Supplement: Supplementary file 1 — Supplementary Information. [file 41598_2020_71169_MOESM1_ESM.pdf]

Supporting Information for '**Downscaling global ocean climate models improves estimates of exposure regimes in coastal environments'**

Fagundes, M.[1,2]; Litvin, S.Y.[3]; Micheli, F.[4,5]; De Leo, G.[4]; Boch, C.A.[3]; Barry, J.P.[3]; Omidvar, S.[1]; Woodson, C.B.[1].

[1]School of Environmental, Civil, Agricultural, and Mechanical Engineering, University of Georgia, Athens, GA 30602, United States of America.

[2]Department of Marine Sciences, University of Georgia, Athens, GA 30602, United States of America.

[3]Monterey Bay Aquarium Research Institute, Moss Landing, California, United States of America.

[4]Hopkins Marine Station, Stanford University, Pacific Grove, California, United States of America.

[5]Stanford Center for Ocean Solutions, Pacific Grove, California, United States of America.

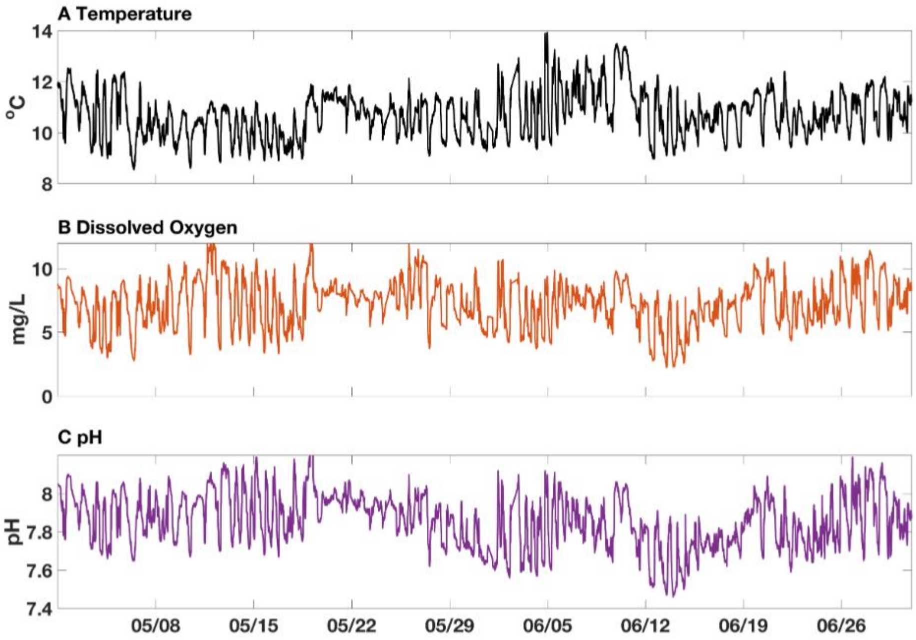

Supplementary Figure 1. Time series of temperature, dissolved oxygen, and pH from Booth et al.[5] showing for a typical period of upwelling during May/June in Monterey Bay.

Model Description

ROMS is a 3-D, free-surface model, which solves the primitive equations (conservation of mass, conservation of momentum, and an equation of state that relates fluid density to temperature and salinity) using hydrostatic and Boussinesq approximation (changes in density are small relative to the overall density; e.g. water density is 1,000 kg m^{-3} and saltwater is 1,030 kg m^{-3}, a 3% difference) with topography-following sigma layers (coordinate system that follows bottom profile and divides water depth accordingly)[42,64]. We ran ROMS in a quasi-3D setup coupled with the biogeochemical model of Fennel[43,44]. The model domain is set to be 30 km in order to allow for any instabilities associated with boundary conditions (BCs) to dissipate. The initial settings for the model are described in Supplementary Table 3. The model has a critical depth (hc) = 20 m, a bottom stretching factor (θ_b) 4.0 (allows increased resolution or smaller grid cells near bottom), and a surface stretching factor (θ_s) 6.5 (allows increased resolution or smaller grid cells near surface). Turbulence/mixing was handled by the Mellor-Yamada 2.5 closure scheme. Horizontal and vertical velocities were initially set to zero. The model domain is set with the coast at the southern boundary (closed boundary) extending northward. The east and west boundaries are set to be periodic so baroclinic waves are not reflected. The northern boundary is forced with barotropic tides estimated from the M1 mooring (Fig. 1 in the main text), and has radiative boundary conditions to allow free propagation of instabilities out of the domain. Temperature and salinity are fixed or nudged to initial conditions. Boundary conditions were set equal to initial conditions and fixed at the northern boundary. East and west boundaries were closed for biogeochemical variables, and set to gradient-free for the northern boundary.

Supplementary Table 1: Initial settings for the Idealized case.

	Model Setup
**Resolution**	50 x 200 m
**dt**	30s
**Sigma Layers**	50
**Atmospheric Forcings**	winds, radiation
**Period of Integration**	3 months
**Output**	Hourly
**Oceanic Forcings ***	M2 =0.062 m/s, K1=0.055 m/s

* Oceanic Forcing estimated from cross-shore barotropic currents at M1 near model. Baroclinic time-step (Dt). Division of the model used in smaller regions (sigma layers).

Atmospheric Forcing

For this idealized experiment, we consider an upwelling background condition and only sea breeze winds. The sea breeze in Monterey Bay is linked to large-scale weather patterns that also drive region-scale upwelling. During strong upwelling periods, the diurnal sea breeze develops over the bay. However, during relaxation of upwelling winds, the diurnal sea breeze is not present (Supplementary Fig. 2).

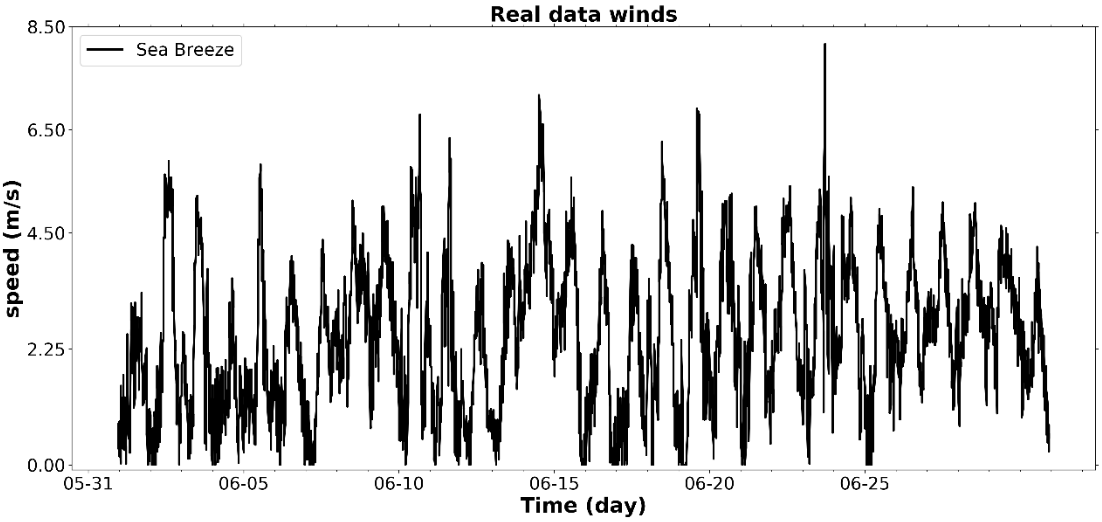

Supplementary Figure 2. Observed diurnal sea breeze winds for southern Monterey Bay.

The upwelling-relaxation cycle in this region is approximately 7 days of upwelling followed by 3 days of relaxation (10-day cycle)[65]. Sea breeze winds were used in order to simulate the movement of warm water lenses. Winds were ramped in the first 20 days of simulation to ensure model stability and both used the same equation to peak during the same periods:

$$\mathrm{V} = \mathrm{A}\sin(\omega_{\mathrm{10d}}\mathrm{t}) + \mathrm{B} \qquad (2)$$

For V > 0:

$$\frac{V(V > 0)}{2}\sin(\frac{-3\pi}{4} + \omega_{24h}t(V > 0)) + \frac{V(V > 0)}{2}$$

$$V = \begin{cases} if < 0 \rightarrow V = 0 \\ if > 0 \rightarrow V = \gamma V \end{cases}$$

where, V is wind velocity, A = 4, and B = 2 are the coefficients used to create the time series. ω_{24h} = 2π/(3600s * 24h) and ω_{10d} = 2π/(10d * 24h * 3600s) are the frequencies for cycles with periods of 24 hours and 10 days, respectively. γ is a coefficient used to match the magnitude of the sea breeze with observations. Thus, negative winds for both situations were considered zero (Supplementary Fig. 3).

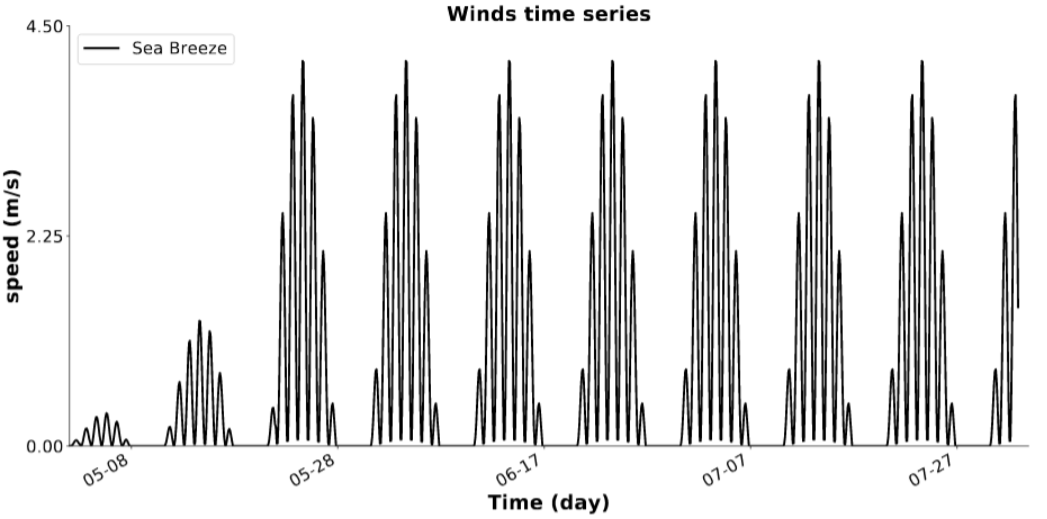

Supplementary Figure 3. Sea breeze and downwelling wind time series used for the simulation.

Solar shortwave radiation (swr) was imposed hourly using a sinusoidal function with varying amplitude depending on the scenarios (Supplementary Fig. 4). The following values were used 150 Watts/m^2, 150 Watts/m^2, 260 Watts/m^2, 260 Watts/m^2, and 300 Watts/m^2 for present, RCP26, RCP45, RCP60, and RCP85, respectively. These values were chosen to prevent the model from losing heat over the period of each run and are all significantly lower than observed values for present day of 600-800 W/m^2 during summer. In addition, air temperature was held at 1°C higher than the maximum surface temperature in the model for each scenario. This prevented the model from losing heat in the surface layers during the simulation. Air pressure was 1013.25 mb for all scenarios. Other atmospheric forcing such as, longwave radiation, rain, cloud was considered zero for the entire experiment since they do not play dominant role during the upwelling period.

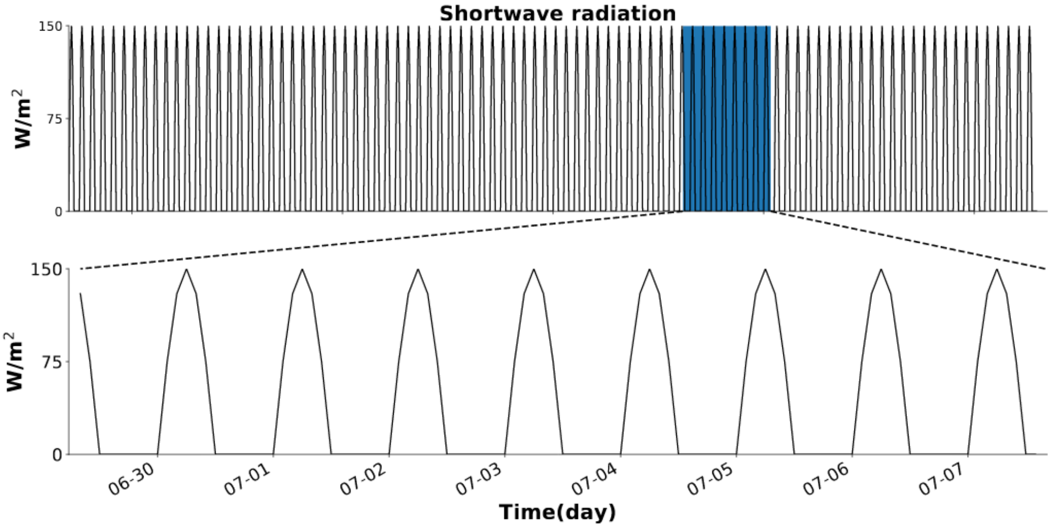

Supplementary Figure 4: Time series used for shortwave radiation.

Ocean Forcing

The model is initialized from rest (all velocities = 0). We represent the tidal cycle by forcing the model at the northern boundary using a meridional (cross-shore) component of velocity with tidal frequencies using:

$$v(z,t) = \begin{cases} 0, if\ z \geq D_{pyc} \\ U_{M2}\cos(\Omega_{M2}t - 1.3\pi) + U_{K1}\cos(\Omega_{K1}t + 1.3\pi)\ otherwise. \end{cases} \quad (3)$$

Where v is the cross-shelf velocity enforced at the northern boundary, U_{M2} and U_{K1} are the velocities, and $\Omega_{M2} = 2\pi/(12.42hr * 3600)$ and $\Omega_{K1} = 2\pi/(23.93hr * 3600)$ are the tidal frequencies, and the depth of the pycnocline (D_{pyc}) was set to 15 m based on observational data. In order to initiate the model, the first four hours of U_{M2} and U_{K1} velocities were set to zero. After the initialization period, U_{M2} and U_{K1} velocities were set to 0.062 m/s, 0.055 m/s, respectively (supplementary Fig. 5). Due to the fact, the model is 2D and therefore does not fully represent the background circulation in the southern region of the Monterey Bay[66], the velocity is set to zero from surface to the pycnocline depth (supplementary Fig. 6). The warm water lenses inside the bay act as a barrier for the tidal currents as they push cold and well mixed waters towards nearshore.

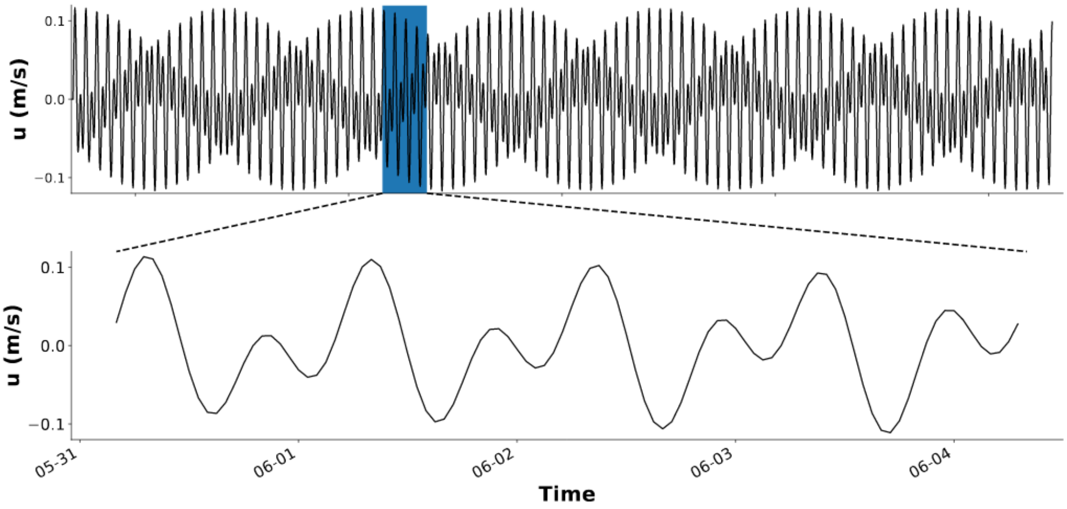

Supplementary Figure 5. Time Series of velocity used to force West boundary in the model.

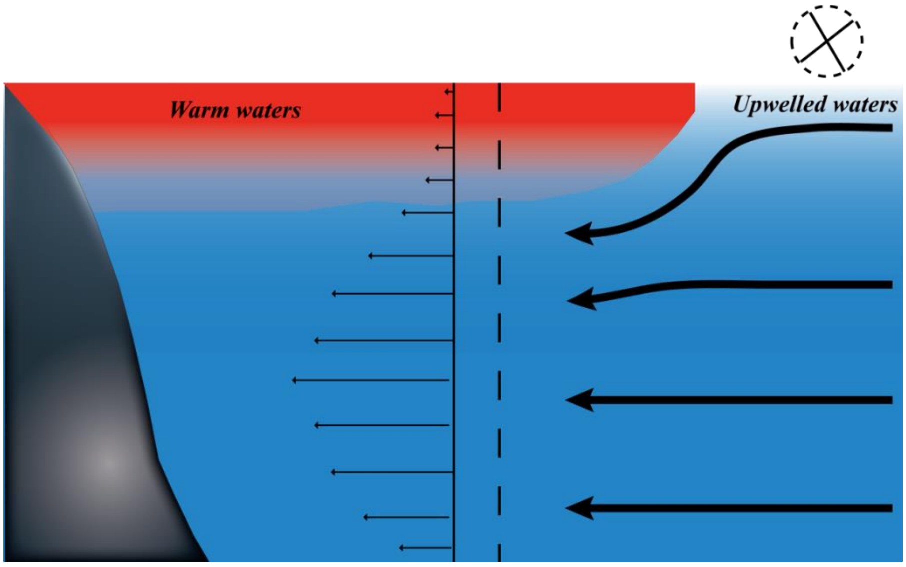

Supplementary Figure 6. Schematic of tidal currents subduction and vertical velocity profile at the site of study. The thicker arrows indicate subtidal currents, the dashed line is the limit of the domain, and the smaller arrows indicate the velocity profile observed with in situ data (not shown) nearshore. The red zones indicate the warm-water of lenses that form during spring and summer seasons[18,32].

Example for Present and Future stressors

For RCP 4.5, we calculated using the following steps:

$$\frac{DO_{Surf}^{Present}}{DO_{Bottom}^{Present}} = 10.0$$

$$\frac{DO_{Surf}^{RCP2.6}}{DO_{Bottom}^{RCP2.6}} = 9.748$$

$$\frac{DO_{Surf}^{RCP8.5}}{DO_{Bottom}^{RCP8.5}} = 15.5$$

$$\mathrm{R} = -0.05377(DO_{Surf}^{RCPs}) + 25.67 \tag{4}$$

Calculating the surface:bottom DO ratio and bottom DO for RCP 4.5:

$$\mathrm{R}(241.7) = -0.05377\mathrm{x}(DO_{Surf}^{RCP4.5}) + 25.67$$

$$DO_{Bottom}^{RCP4.5} = \frac{DO_{Surf}^{RCP4.5}}{R(241.7)}$$

Supplementary Table 2. Adjusted values for surface temperature and surface and bottom oxygen in present and future scenarios found by fitting the eqn. 1 and used as Initial and Boundary conditions for the Idealized experiment to account for increased oxygen in surface layers due to kelp.

Scenarios	Location	T (°C)	O_2(mmol m^{-3})	O_2(mg L^{-1})
Present	Surface		360	11.25
	Bottom	8	93.24	2.91
RCP 2.6	Surface		303.7	9.50
	Bottom	10.28	69	2.16
RCP 4.5	Surface		301.7	9.43
	Bottom	10.35	68	2.13
RCP 6.0	Surface		296.7	9.27
	Bottom	10.44	65.2	2.03
RCP 8.5	Surface		292.8	9.15
	Bottom	10.83	63	1.97

Supplementary Table 3. Parameters used in logistic regression equations 5 and 6. Davies Test Breaking-Point estimation[67]. $\beta_0 = \beta_{pH}$ = Intercept; β = Slope Segment 1; β_2 = Slope Segment 2; β_A = Slope (pH x Temperature Group); β_B = Slope (high temperature); BP = Breaking Point.

**Parameters**	Temp. $\leq 13°C$	Temp. $> 18°C$
β_0	-32.78	-20.83
β_{ph}	4.28	2.67
β	4.29	
β_2	1.18	
β_A		-2.13
β_B		16.75
**Davies test BP estimate**	7.56	
**Offset**	9.344	

Model Results

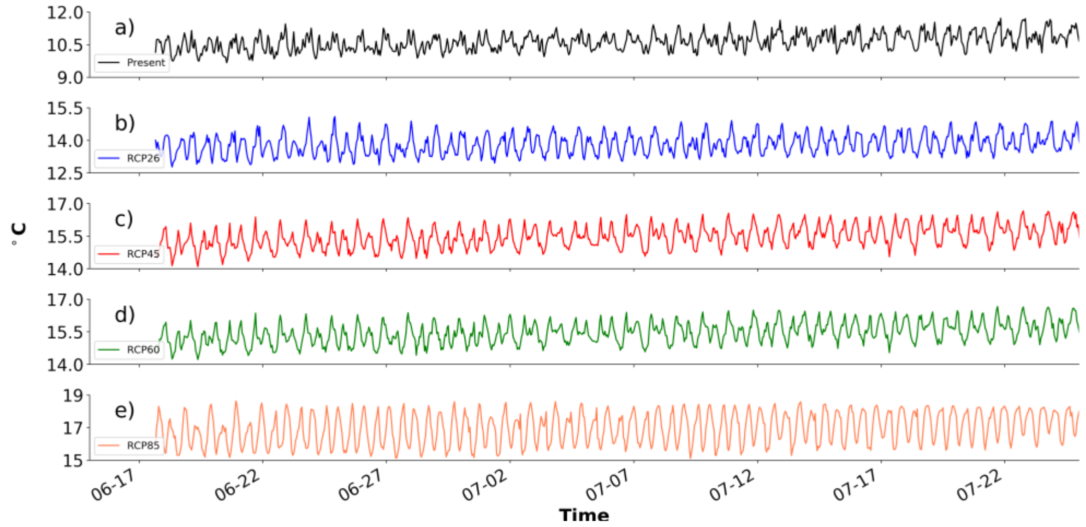

Supplementary Figure 7, Modeled time series of Temperature for 1-month period with idealized upwelling for present day (a), RCP2.6 (b), RCP4.5 (c), RCP6.0 (d), and RCP8.5 (e).

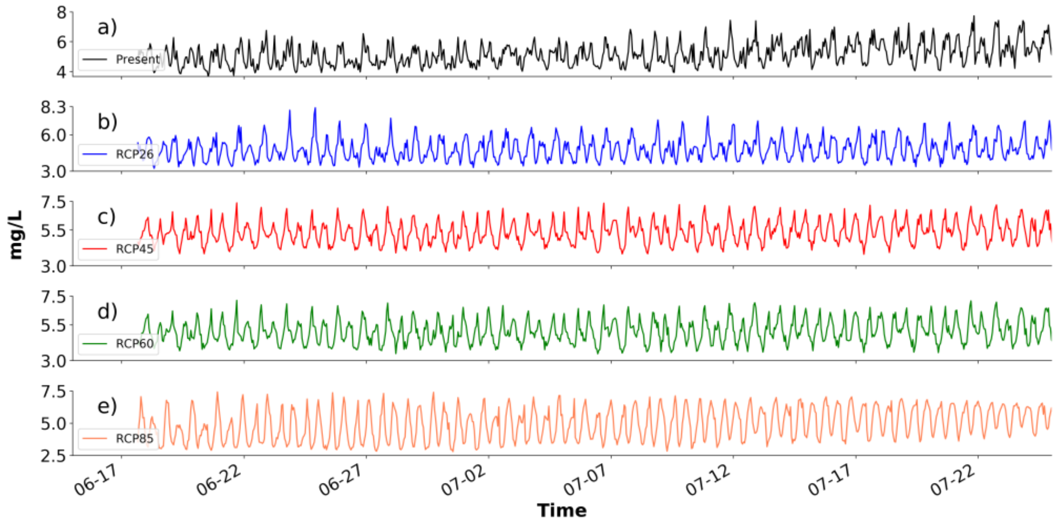

Supplementary Figure 8. Modeled time series of O_2 for 1-month period with idealized upwelling resent day (a), RCP2.6 (b), RCP4.5 (c), RCP6.0 (d), and RCP8.5 (e).

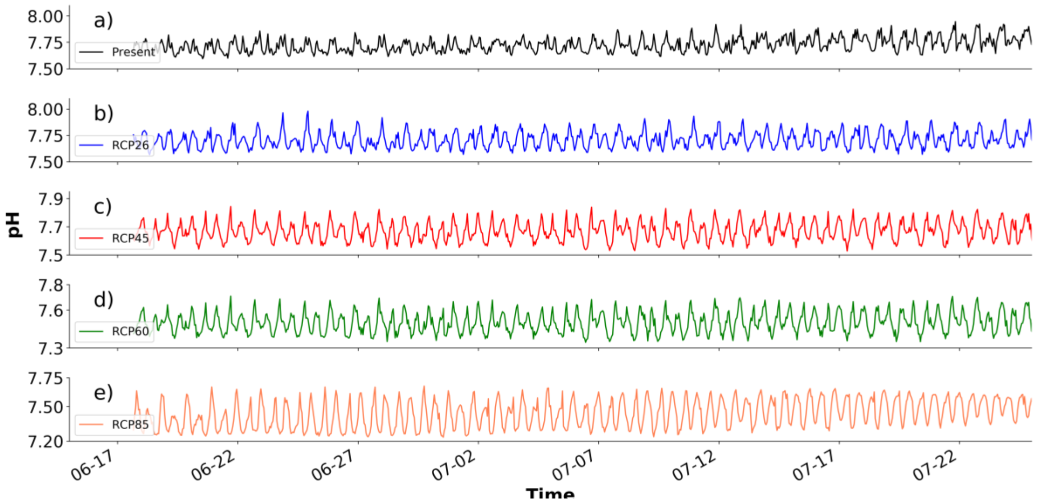

Supplementary Figure 9.` Modeled time series of pH for 1-month period with idealized upwelling Present day (a), RCP2.6 (b), RCP4.5 (c), RCP6.0 (d), and RCP8.5 (e).

Climate Change Variability

In general, daily variability in temperature, DO, and pH increased as pCO_2 increased in the atmosphere. The intensity as well as daily variability were most pronounced in RCP 8.5 when compared to present for all variables (Supplementary Fig. 10). Differences in temperatures were observed starting from RCP 4.5. The temperature variability range was the smallest in Present and does not change much for RCP 2.6, RCP 4.5, and RCP 6.0.

DO variability in Present is significantly different from RCP scenarios 2.6 through 6.0 (Supplementary Fig. 10b). The median daily variability for RCP 2.6 through 6.0 found was as high as the maximum daily variability in Present. We did not observe any difference when comparing the 3 scenarios against each other by their range.

The variability for pH also showed increasing trends in with higher RCP scenarios (Supplementary Fig. 10c). Present up to RCP 6.0 scenarios were not significantly different from each other. However, a notably difference was seen when comparing present and RCP 8.5. Moreover, the range of the variability also doubled when comparing present and RCP 8.5 scenarios.

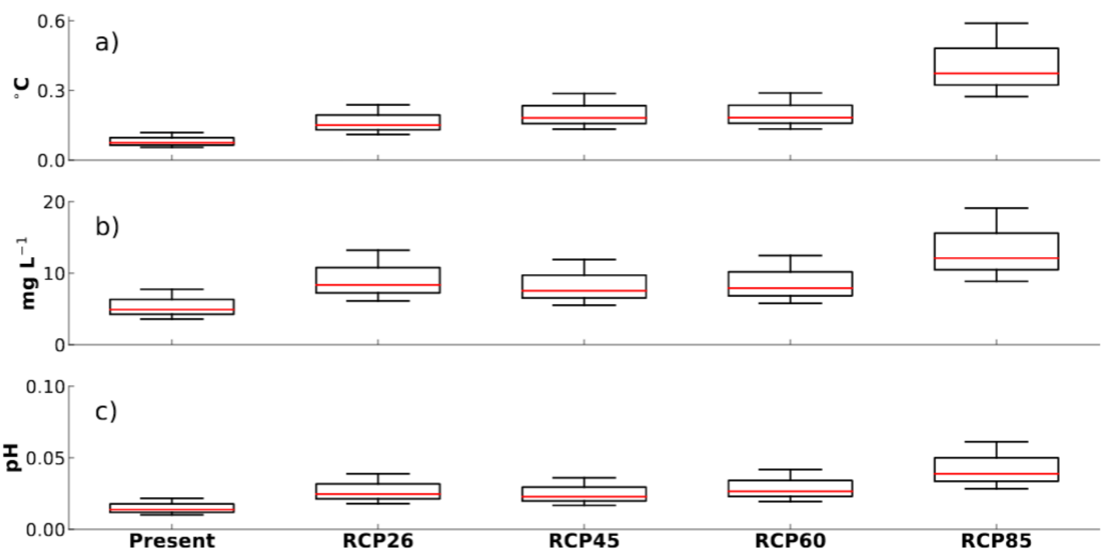

Supplementary Figure 10: Integrated daily variability for stressors: Temperature, DO, and pH. The y-axis gives the a) temperature, b) DO, and c) pH and the x-axis represents the scenarios. Red lines give the median of temperature, DO, and pH for each scenario. Whiskers represent the 95% confidence interval estimated using spectral analysis.

References

64. Shchepetkin, A. F. & McWilliams, J. C. The regional oceanic modeling system(ROMS): A split-explicit, free-surface, topography-following-coordinate oceanic model. *Ocean Model.* 9, 347–404 (2005).

65. Graham, W. M. & Largier, J. L. Upwelling shadows as nearshore retention sites: The example of northern Monterey Bay. *Cont. Shelf Res.* 17, 509–532 (1997).

66. Suanda, S. H., Barth, J. A. & Woodson, C. B. Diurnal heat balance for the northern Monterey Bay inner shelf. *J. Geophys. Res. Ocean.* **116**, 1–13 (2011).

67. Muggeo, V. R. M. segmented: An R Package to Fit Regression Models with Broken-Line Relationships. *R News* **3**, 343–4 (2008).
